# ABO and RhD blood group distributions in Gansu Province, China: A retrospective study based on donor and patient data

**DOI:** 10.1097/MD.0000000000049048

**Published:** 2026-05-29

**Authors:** Xiang Zhang, Miaomiao Liu, Kang Liu, Yigang He

**Affiliations:** a Department of Blood Transfusion Medicine, The 940th Hospital of the Joint Logistics Support Force, Lanzhou, Gansu Province, China; b Department of Diagnostic Radiology, The 940th Hospital of the Joint Logistics Support Force, Lanzhou, Gansu Province, China.

**Keywords:** ABO blood groups, blood supply–demand equilibrium, blood group distribution, RhD blood groups

## Abstract

The distribution of ABO and Rhesus D (RhD) blood groups is crucial for ensuring the safety of blood transfusions and for optimal blood donation planning. Gansu Province in Northwest China is characterized by remarkable ethnic diversity, which may contribute to a unique blood group distribution pattern in this region. This study investigates blood group distribution in Gansu Province, China, and considers the implications for the balance between blood supply and demand. Data from 71,363 blood donors (2014–2023) and 188,827 patients (2018–2023) were extracted from the hospital information management system. The chi-square test was used to compare blood group distributions between the 2 populations, and linear regression was performed to evaluate temporal trends in donation volumes. All analyses and graphing were performed using GraphPad Prism 5. The overall distribution of ABO and RhD blood groups differed significantly between donors and patients (*P* < .001). The prevalence of O (RhD+) was significantly lower in patients than in donors (30.54% vs 32.22%, *P* < .001), while the proportions of A (RhD+) and B (RhD+) were significantly higher in patients (A: 28.46% vs 27.66%, *P* < .001; B: 30.83% vs 30.02%, *P* = .001). For RhD-negative individuals, the proportion of AB (RhD−) in patients (0.06%) was double that in donors (0.03%, *P* = .016). Linear regression analysis confirmed a significant downward trend in donation volumes from 2018 to 2023 (β = −581.0 units/yr, 95% confidence interval: −1060 to −102, *R*^2^ = 0.76, *P* = .018). Total red blood cells demand consistently exceeded supply. And the supply–demand gap was further widened by discrepancies in the blood type distribution between donors and patients. This study reveals significant differences in ABO/RhD distributions between donors and patients in Gansu Province. The supply–demand imbalance is driven primarily by insufficient total donations and is further aggravated by blood type mismatches between donors and patients.

## 1. Introduction

The term “blood group” is generally used to describe a set of antigen combinations expressed on the surface of blood cells. To date, the International Society of Blood Transfusion has catalogued 48 different blood group systems covering 371 different types of red cell antigens.^[1]^ Of these, the ABO and Rhesus D (RhD) blood groups have the greatest clinical significance.^[2]^ The distribution of ABO and RhD blood groups varies among populations in different countries and regions.^[3–6]^ Timely knowledge of the distribution of local blood groups is necessary because it not only helps to ensure the safety of blood transfusion but also helps to establish a more precise blood donation plan to maintain a balance between blood supply and demand.

Gansu Province, a transportation hub and tourist destination in northwestern China, is characterized by remarkable ethnic diversity. According to the Seventh National Population Census (2020), ethnic minorities constitute 10.62% of the province’s 25.02 million population, and Gansu is home to 55 of China’s 56 ethnic groups – the highest diversity among all Chinese provinces. Previous studies have documented significant interethnic variations in ABO and RhD blood group frequencies in China,^[6]^ suggesting that this demographic uniqueness may contribute to a distinctive regional blood group distribution pattern.

Recent research has examined the regional distribution of ABO blood groups across China, including Gansu Province.^[3]^ However, that study did not address RhD distribution and relied exclusively on blood donor data. Consequently, whether blood group distribution among patients – those who actually receive transfusions – differs from that of donors remains unexplored.

This study aimed to characterize ABO and RhD blood group distributions in Gansu Province through a comprehensive analysis of both donor and patient data. By comparing distributions between these 2 populations, we sought to identify potential discrepancies that could inform more rational blood donation planning. In addition, by analyzing temporal trends in blood supply and demand for different blood types, we aimed to assess supply–demand gaps and provide evidence for developing more effective donation programs and inventory management strategies to meet clinical needs in this ethnically diverse region.

## 2. Materials and methods

In this study, ABO and RhD blood group data were extracted from the hospital information management system for blood donors (2014–2023) and patients (2018–2023). Duplicate records were removed using unique national identification numbers for donors and unique medical record numbers for patients. Blood group distributions were analyzed separately. Annual comparisons between donor and patient data from 2018 to 2023 were performed to assess supply–demand differences for each blood group. The chi-square test was used to compare blood group distributions between the 2 populations, and linear regression was performed to evaluate temporal trends in donation volumes. All analyses and graphing were performed using GraphPad Prism (version 5.0; GraphPad Software, San Diego). A *P* value of <.05 was considered statistically significant.

This study was conducted in accordance with the Declaration of Helsinki and approved by the Ethics Committee of the 940th Hospital of the Joint Logistics Support Force.

## 3. Results

### 3.1. Blood group distribution analysis

A total of 71,363 blood donors (2014–2023) and 188,827 patients (2018–2023) were included. The overall distribution of ABO and RhD blood groups differed significantly between the 2 populations (χ^2^ = 267.4, *df* = 7, *P* < .001). Detailed distributions are shown in Table 1.

**Table 1:** Distribution of ABO and RhD blood groups among donors and patients.

Blood group	Donors (n = 71,363)	Patients (n = 188,827)	*P* value
A RhD+	19,741 (27.66%)	53,731 (28.46%)	<.001
B RhD+	21,426 (30.02%)	58,224 (30.83%)	.001
O RhD+	22,995 (32.22%)	57,671 (30.54%)	<.001
AB RhD+	6844 (9.59%)	18,137 (9.61%)	.888
A RhD−	86 (0.12%)	293 (0.16%)	.039
B RhD−	135 (0.19%)	308 (0.16%)	.141
O RhD−	113 (0.16%)	358 (0.19%)	.102
AB RhD−	23 (0.03%)	105 (0.06%)	.016

RhD = Rhesus D.

Among RhD-positive individuals, the most common blood group in both cohorts was O, followed by B, A, and AB. However, notable differences were observed for specific groups. The prevalence of O (RhD+) was significantly lower in patients than in donors (30.54% vs 32.22%, *P* < .001), whereas the proportions of A (RhD+) and B (RhD+) were significantly higher in patients (A: 28.46% vs 27.66%, *P* < .001; B: 30.83% vs 30.02%, *P* = .001). The frequency of AB (RhD+) was similar between the 2 groups (9.61% vs 9.59%, *P* = .888).

RhD-negative individuals constituted a small fraction of both cohorts (0.57% in patients vs 0.50% in donors). Among these, the proportion of A (RhD−) was significantly higher in patients (0.16% vs 0.12%, *P* = .039). The most striking difference was observed for AB (RhD−), where the proportion in patients (0.06%) was double that in donors (0.03%, *P* = .016). No significant differences were found for B (RhD−; 0.16% vs 0.19%, *P* = .141) or O (RhD−; 0.19% vs 0.16%, *P* = .102).

### 3.2. Dynamic changes in red blood cell supply and demand by blood group

**Figure 1.**
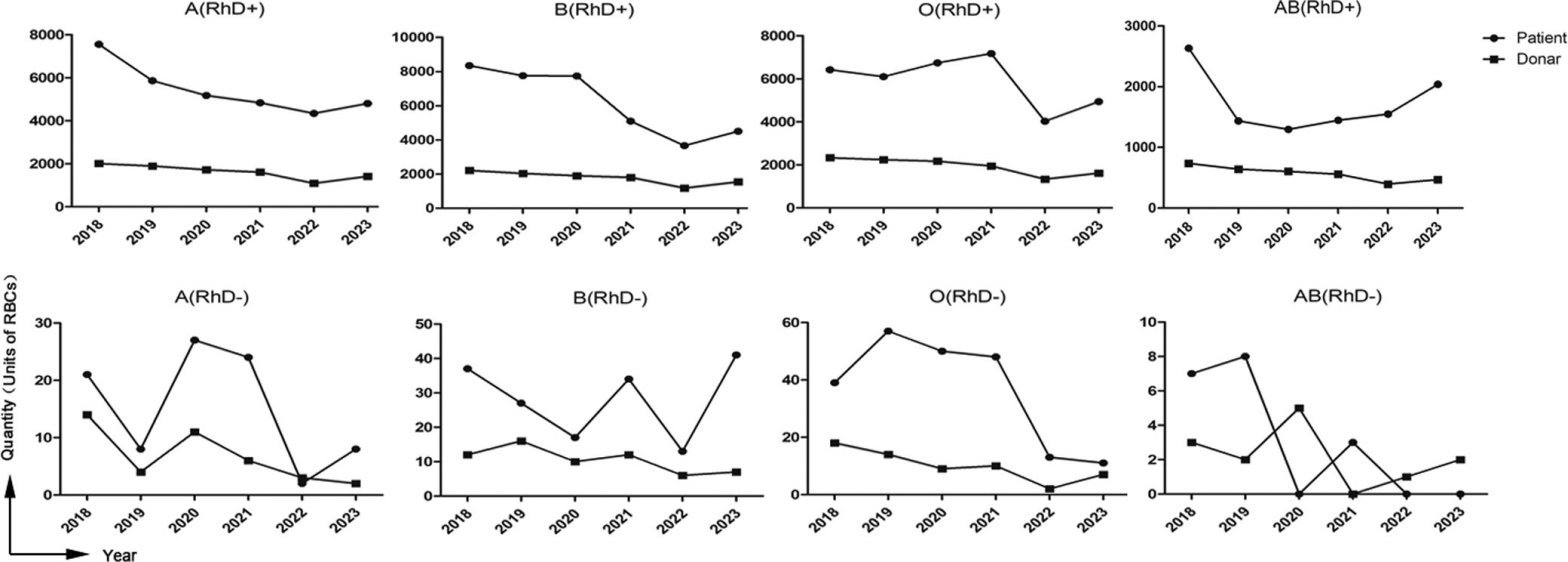
Absolute quantities of annual demand and supply for ABO/RhD blood types (2018–2023). The figure shows the yearly absolute number of RBC units demanded by patients and the corresponding supply for each of the 8 ABO/RhD blood types from 2018 to 2023. The gap between demand and supply visually represents the supply–demand mismatch. RBC = red blood cell, RhD = Rhesus D.

The observed discrepancies in blood group distributions between donors and patients suggest a potential imbalance in blood supply and demand for different blood types. To investigate this, a dynamic annual analysis of red blood cell (RBC) supply and demand was conducted from 2018 to 2023, with results shown in Figure 1.

Throughout the study period, total RBC demand consistently exceeded supply, and donation volumes showed a declining trend each year. Linear regression analysis confirmed a statistically significant downward trend in donation volumes (β = −581.0 units per year, 95% confidence interval: −1060 to −102, *R*^2^ = 0.76, *P* = .018). A partial recovery was observed in 2023, but donation levels remained below pre-pandemic levels.

**Figure 2.**
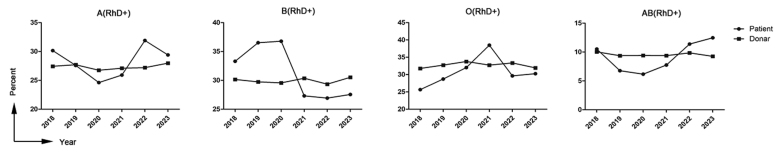
Comparative relative frequencies of ABO/RhD blood types between patient demand and donor supply (2018–2023). This normalized view shows the annual relative frequency (percentage) of each of the 8 ABO/RhD blood types for patient demand versus donor supply from 2018 to 2023, enabling a direct comparison of the distribution structure between the 2 groups. RhD = Rhesus D.

As demand far exceeded supply in absolute terms, the data were converted into percentages for relative comparison to better understand how blood type mismatches contribute to the supply–demand imbalance. These results are shown in Figure 2. The proportional analysis revealed persistent differences between patients and donors across blood groups over time, which, under conditions of overall shortage, undoubtedly exacerbate the imbalance. It should be noted that the proportion of RhD-negative RBCs was relatively low and was therefore excluded from percentage conversion.

## 4. Discussion

Regional differences in blood group distribution are well documented and are primarily attributed to variations in ethnic and genetic backgrounds. For example, a study in Chongqing Municipality reported ABO blood group frequencies based on donor data of A: 31.72%, B: 24.02%, O: 35.36%, and AB: 8.36%,^[7]^ which differ markedly from our donor-derived estimates for Gansu (A: 27.78%, B: 30.21%, O: 32.38%, AB: 9.62%). Both sets of estimates are derived from blood donor populations, indicating that the observed regional disparities reflect genuine differences in population genetic composition rather than methodological variations. These differences are likely attributable to distinct demographic compositions. Chongqing’s population is predominantly Han Chinese (approximately 95%), with Tujia (2.5%) and other minorities collectively accounting for the remaining 2.5%.^[7]^ In contrast, according to the Seventh National Population Census (2020), Gansu has a more diverse ethnic makeup: Han Chinese constitute 89.0% of the population, followed by Hui (5.3%), Tibetan (2.0%), and other minority groups. Previous studies have documented significant inter-ethnic variations in ABO and RhD blood group frequencies in China,^[6]^ suggesting that Gansu’s greater ethnic diversity contributes to its distinctive blood group distribution pattern.

Current global research on blood group distribution is primarily based on data from blood donors. However, blood donors are subject to strict eligibility criteria (e.g., age, weight, hemoglobin levels) and therefore represent a selected population that may not fully reflect the general population. In contrast, patient data include individuals of all ages seeking medical care for a wide range of diseases and injuries, with no such eligibility restrictions, thereby providing a complementary sample for inferring population blood group distribution. In the present study, we observed significant differences in blood group distributions between donors and patients, particularly for O (RhD+), A (RhD+), B (RhD+), and AB (RhD−). This suggests that population estimates based solely on donor data may be biased, and the inclusion of patient data offers an independent reference for comparison. A similar methodological approach has been used internationally; for example, Australian researchers analyzed blood group distribution using data from both blood donors and patients.^[8]^ Yang et al analyzed the distribution characteristics of ABO blood groups nationwide.^[3]^ Their results for Gansu Province (A: 27.97%, B: 30.60%, O: 32.06%, AB: 9.36%) are generally consistent with our donor-based estimates (A: 27.78%, B: 30.21%, O: 32.38%, AB: 9.62%). This consistency suggests that our donor data are reliable and comparable with previous regional estimates. Building on this baseline, the significant differences observed between donors and patients in our study are unlikely to stem from sampling error, but rather reflect genuine differences between these 2 populations.

From the blood supply–demand perspective, a clear imbalance between supply and demand was observed throughout the study period. Patient demand data can provide a basis for blood collection agencies to develop more rational plans. The results showed that both blood demand and supply exhibited declining trends from 2018 to 2023, with the most pronounced decrease occurring between 2019 and 2022. The lowest point of RBC demand and supply was in 2022, which was likely influenced by the COVID-19 pandemic. Several studies have documented significant reductions in blood collection during pandemic lockdowns in China, attributed to restricted mobility, closure of donation sites, and fear of infection among potential donors.^[9,10]^ The partial recovery observed in 2023 suggests a gradual return to pre-pandemic donation behavior, although donation levels have not yet fully rebounded. Notably, the demand for AB (RhD+) blood showed a consistent upward trend throughout the study period, and whether this trend will continue needs to be verified with subsequent data.

For RhD-negative RBCs, overall demand also exceeded supply. The finding that AB (RhD−) proportion in patients (0.06%) was double that in donors (0.03%, *P* = .016) has important clinical implications. As one of the rarest blood types, this disparity suggests patients with AB (RhD−) may have disproportionately higher transfusion needs relative to their donor representation. Given the limited data, these findings should be interpreted with caution. Nevertheless, establishing a comprehensive rare blood donor registry and developing frozen red blood cell banks may help improve timely transfusion access for these patients.

## 5. Conclusion

This study provides a comprehensive analysis of ABO and RhD blood group distributions in Gansu Province using both donor and patient data. Significant differences were observed between the 2 populations. While the primary supply–demand challenge remains insufficient total donations, blood type mismatches between supply and demand further exacerbate this imbalance. These findings update the population blood group distribution for Gansu and provide evidence for developing targeted donation strategies and improving institutional blood inventory management, such as establishing frozen red blood cell banks to mitigate supply–demand fluctuations.

## Author contributions

**Conceptualization:** Xiang Zhang, Yigang He.

**Software:** Xiang Zhang.

**Data curation:** Miaomiao Liu.

**Funding acquisition:** Kang Liu, Xiang Zhang.

**Visualization:** Kang Liu.

**Writing – review & editing:** Kang Liu, Yigang He.

**Writing – original draft:** Xiang Zhang.
